# The MAIN Shirt: A Textile-Integrated Magnetic Induction Sensor Array

**DOI:** 10.3390/s140101039

**Published:** 2014-01-09

**Authors:** Daniel Teichmann, Andreas Kuhn, Steffen Leonhardt, Marian Walter

**Affiliations:** 1 Philips Chair for Medical Information Technology, RWTH Aachen University, Pauwelsstr. 20, Aachen 52074, Germany; E-Mails: leonhardt@hia.rwth-aachen.de (S.L.); walter@hia.rwth-aachen.de (M.W.); 2 Euro Engineering AG, Karlsruhe 76135, Germany; E-Mail: Andreas.Kuhn@rwth-aachen.de

**Keywords:** textile integration, magnetic induction, cardiorespiratory monitoring, mobile devices

## Abstract

A system is presented for long-term monitoring of respiration and pulse. It comprises four non-contact sensors based on magnetic eddy current induction that are textile-integrated into a shirt. The sensors are technically characterized by laboratory experiments that investigate the sensitivity and measuring depth, as well as the mutual interaction between adjacent pairs of sensors. The ability of the device to monitor respiration and pulse is demonstrated by measurements in healthy volunteers. The proposed system (called the MAIN (magnetic induction) Shirt) does not need electrodes or any other skin contact. It is wearable, unobtrusive and can easily be integrated into an individual's daily routine. Therefore, the system appears to be a suitable option for long-term monitoring in a domestic environment or any other unsupervised telemonitoring scenario.

## 1. Introduction

Telemonitoring might enable the elderly and/or ill persons to live longer in their individual domestic environment. This would considerably increase their quality of life, as well as decrease the costs for national health systems [1]. In particular, persons needing frequent medical control, but living, for example, in a rural area some distance from a medical institution would benefit from such a system.

Telemonitoring systems that are practicable for this type of scenario require smart sensor-solutions for long-term monitoring of vital signs. Moreover, appropriate smart sensor techniques have to meet several requirements. For example, the application should be as easy as possible, *i.e.*, the effort required to position the sensor has to be reduced to a minimum. The probability of forgetting to apply the sensor should also be minimized; this is particularly important for elderly persons. Furthermore, to avoid skin irritation, the use of electrodes is not desirable. Finally, the limitation of a patient's mobility should be avoided by the use of mobile devices and wireless data transmission.

The presented MAIN Shirt (MAIN: short for magnetic induction) comprises four non-contact sensors based on electromagnetic coupling between a coil and the thorax, basically due to magnetic eddy current induction. The preliminary results of a prototype are published in [2,3]. Since magnetic induction monitoring is a non-contact monitoring technique, it fulfills most of the requirements described above. Moreover, since the sensors are integrated into a shirt, positioning is no longer an issue. Furthermore, integration into clothing minimizes the risk of forgetting to apply the sensor.

Common techniques for magnetic eddy current induction measurements are based on a multiple coil method [4–8] using one coil for field excitation and further ones for field measurement. Since these multiple coils have to be precisely calibrated in a geometric alignment, this setup is not suitable for textile integration. Therefore, to allow the possibility of a flexible sensor coil, a method based on a single coil is used (described in detail in Section 2.1). This method was first introduced by Vas *et al.* in 1967 [9]. Over the last three decades, the method has occasionally been investigated by four research groups [10–13]. In addition, a non-wearable system for bedside respiratory monitoring was presented that was also based on a flexible coil [14].

For the purpose of mobility and integration into the shirt, the measurement circuitry of the sensors is reduced to a minimum by using a frequency modulation approach incorporating a digital frequency counter. A similar (but rigid) system for use on a chair was introduced by our research group in [15]. To the authors' knowledge, the system described here is the first wearable and textile-integrated version of a magnetic eddy current induction or electromagnetic coupling sensor. The system's ability to reliably assess respiration and pulse was evaluated by means of measurements in healthy volunteers.

## 2. Methods

The sensor part of the device is based on magnetic induction (the physical principle is described in Section 2.1). The integration of the sensors into the shirt is described in Section 2.2. To evaluate cardiac monitoring during motion, the raw signal of the system was processed and analyzed by discrete wavelet transform (DWT) (Section 2.4).

### 2.1. Sensing Principle

Magnetic induction monitoring is a non-contact technique to assess cardiorespiratory activity by measuring the impedance distribution within the thorax.

A sensor coil is driven by an alternating current, so that it excites an alternating magnetic field, ***B*_1_**. ***B*_1_** induces eddy currents within the thorax. These eddy currents, in turn, excite another alternating magnetic field, ***B*_2_**, whose size and orientation depends on the thoracic impedance distribution. By combining the Biot-Savart law and the law of induction, the elementary component, ***B*_2_**, of the reinduced field caused by the elementary eddy current loop within the field of eddy currents can be calculated by:

(1) $$B_{2}=-\frac{\mu }{4\pi }\frac{1}{Z_{\text{body}}}\frac{d\Phi_{1}}{dt}\int_{\partial s_{2}}dl_{2}\times \frac{r_{2}}{{\left|r_{2}\right|}^{3}}$$

where *μ* denotes the magnetic permeability, Φ_1_ denotes the magnetic flux, *∂S*_2_ describes the boundary curve corresponding to the area, *S*_2_, spanned by the elementary eddy current path, *Z_body_* denotes the thoracic impedance along the eddy current path, *d****l*_2_** denotes the infinitesimal length of the current path tangential to the curve, *∂S*_2_, and ***r*_2_** is the displacement vector between *d****l*_2_** and a point in the environment.

Since the distribution of the thoracic impedance varies with physiological activity (described below), the ***B*_2_**-field also varies. Therefore, information on cardiorespiratory activity can be obtained by measuring the variation of ***B*_2_**. A common way to measure ***B*_2_** is to monitor the voltage, *U_meas_*, induced by ***B*_2_** into a separate measurement coil (voltage induction by ***B*_1_** is eliminated as described below) according to the law of induction:

(2) $$U_{\text{meas}}=\frac{\mu }{4\pi }\int_{S_{\text{meas}}}\int_{S_{2}}\int_{\partial S_{2}}\frac{1}{Z_{\text{body}}}\frac{d^{2}{\text{B}}_{1}}{dt^{2}}dl_{2}\times \frac{r_{2}}{{\left|r_{2}\right|}^{3}}dS_{2}dS_{\text{meas},}$$

where *S_meas_* denotes the area of the measurement coil.

**Figure 1.**
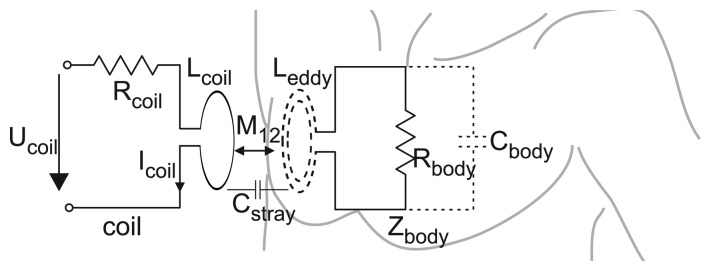
Sensing principle. The magnetic field sent out by the sensor coil induces thoracic eddy currents, which reinduce a secondary magnetic field affecting the reflected impedance of the coil. As shown, this resembles a transformer model.

To eliminate disturbance by the primary magnetic field, the measurement coil has to be precisely calibrated by a geometrical alignment (orthogonal or gradiometric arrangements) [7,8,16]. To overcome the necessity of this geometrical alignment and its problems regarding flexibility, the sensors integrated in the MAIN Shirt follow a slightly different approach using a single coil for field excitation and for field measurement. This is based on the fact that the reinduced field, ***B*_2_**, affects the primary one, ***B*_1_**, and, hence, changes the so-called reflected impedance of the coil [17]. A way of describing this measurement principle is to regard the sensor coil as the primary coil and the eddy currents in their collective as the secondary coil of a traditional transformer model with mutual inductance *M*_12_ (see Figure 1) [10,17,18].

The load of the secondary coil consists of a conductive part (*R_body_*) and a capacitive part (*C_body_*) that model tissue conductivity and permittivity, respectively Using this model and applying analytical signals (here, sinusoidal), the complex reflected coil impedance can be expressed by [15]:

(3) $$Z_{\text{coil},r}=\frac{U_{\text{coil}}}{I_{\text{coil}}}=R_{\text{coil}}+j\omega L_{\text{coil}}-\frac{\omega^{3}C_{\text{body}}M_{12}^{2}}{j\omega C_{\text{body}}(R_{\text{body}}+j\omega L_{\text{eddy}})+1}$$

The stray capacitance (*C_stray_*) between the coil and thorax can be neglected, as long as the distance between the coil surface and the thoracic wall is constant.

There are three different ways in which physiological activity (*i.e.*, respiration and pulse) can modulate the reflected impedance of the coil enabling vital sign monitoring:

- Thoracic impedance (volume change, boundary displacement)
  The volumes of organs, tissue or blood can be varied by physiological activity This may change the conductivity within a specific body region and influence the current strength. Furthermore, the displacement of inner and outer boundaries of organs changes the direction of current paths and, hence, the resistivity along the current path.
- Distance
  A variation in the distance between the sensor and the thoracic wall modifies the magnetic coupling factor, *k*, and, also, the amount of parasitic capacitive coupling, *C_stray_*
- Deflection
  A change in the form of the coil (by bending) or the eddy current paths (by organ displacement) would change *k*, as well as the primary (*L_coil_*) or secondary (*L_eddy_*) self-inductance.

To keep the measurement circuitry as small as possible, an indirect measurement method based on frequency modulation has been chosen to derive the reflected impedance of the coil. Using this method, the coil is a frequency determining part of an oscillatory circuitry. Hence, the oscillatory frequency equals the sending frequency of the magnetic field sent out by the coil. When the impedance of the coil changes due to cardiorespiratory activity, it produces a change of the oscillatory frequency, which can be measured by a frequency counter [13].

### 2.2. System Realization

**Figure 2.**
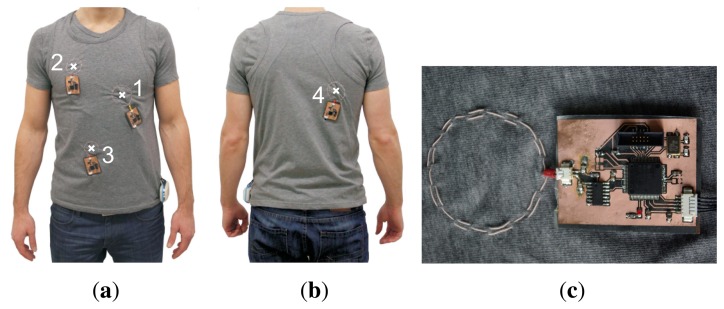
System realization. Three sensors are located at (a) the front and one at (b) the back of the thorax (coil centers are marked); (c) a close-up view of a single sensor.

The MAIN Shirt comprises a sensor array of four magnetic induction sensors. Three are located at the front and one on the back of the shirt (Figure 2a and 2b). Sensor 1 is located left of the apex for optimal pulse monitoring. Sensors 2–4 are located on the chest, abdomen and back in order to cover the best position for respiratory monitoring in different postures. Table 1 presents data on the radius, number of windings and the tuned working frequency of each coil.

As shown in Figure 2c, the sensor head (coil) of each sensor is realized by high frequency litz wire that is sewn into the shirt. The litz wire consists of 460 wire strands and has a diameter of 0.2 mm. The coil is part of a Colpitts oscillator. The frequency change due to a variation in coil impedance can be measured by a frequency counter realized by using the counter input of a microcontroller (MSP430F5435A, Texas Instruments, Dallas, TX, USA) as described in [15]. The oscillator together with the microcontroller is placed on a flexible, one side-printed circuit board (with a physical dimension of 37 × 50 mm).

**Figure 3.**
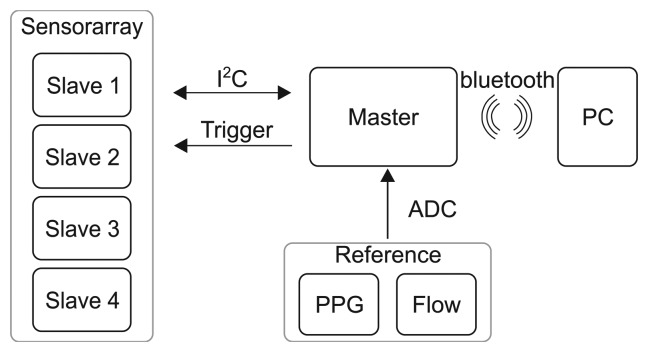
System overview of the MAIN (magnetic induction) Shirt.

The microcontrollers included in each sensor are also used to perform data communication using the I^2^C protocol. The four sensors are configured as slaves and send their data to a master module, which gathers all data and transmits it to a PC *via* Bluetooth (Figure 3). The master module is placed in a small plastic housing located at the waist and includes the Bluetooth-transmitter and a lithium-polymer battery. To ensure synchronicity between the sensor signals, the master unit triggers the sensor units measurements. There are a total of four lines between the master and each slave unit (data, supply, ground, triggering), which are also sewn into the shirt. Data are obtained with a sampling interval of 10.5 m and a frequency counter resolution of 3.125 Hz. The whole system is battery-driven with a supply voltage of 3.7 V and a maximum power consumption of 0.62 watts (0.37 watts is required by the Bluetooth transmitter).

### 2.3. Experimental Sensor Characterization

#### 2.3.1. Sensor Response and Measuring Depth

**Figure 4.**
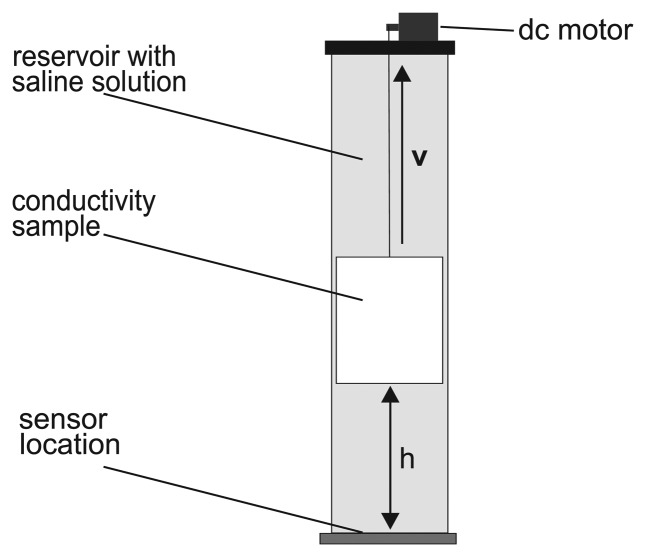
Measurement setup for the examination of sensor response and measuring depth.

As described above, there are various ways in which physiological activity can influence the sensor signal. To investigate the coupling between the sensor and conductive objects, and the influence of the coil parameters, *i.e.*, radius r, number of windings *n* and tuned base frequency *f*_0_, the measurement setup illustrated in Figure 4 was built. The sensor coil was placed beneath a cylindrical reservoir filled with saline solution. In the middle of this reservoir is a channel that serves as a check rail for another, smaller tank (sample) filled with saline solution, whose conductivity, *σ_sample_*, was varied during the experiment. The diameters of the reservoir and the sample tank were 8.5 and 4.5 cm, respectively. Both were made of polypropylene. The sample tank was weighted with pebble stones and could be lifted by a DC motor. The sample conductivity ranged from *σ_sample_* = 1 ms/cm to *σ_sample_* = 8 ms/cm to represent different organ and tissue types (see Table 2) [19-21]. The conductivity of the saline solution within the cylindrical reservoir acting as the surrounding tissue was *σ_tank_* = 1 ms/cm. This allowed us to compare the change of the sensor signal due to object motion with the change due to object conductivity. Furthermore, the measurement setup was used to examine the measuring depth, *i.e.*, the maximum distance, *h*, where the magnetic field penetrating into the reservoir is still high enough for the detection of sample motion.

#### 2.3.2. Mutual Interaction between Adjacent Coils

Another important point when using a sensor array is the mutual interaction between adjacent coils. The principle of magnetic induction monitoring is based on the superposition of an exciting primary magnetic field and a reinduced secondary magnetic field (see Section 2.1). When using several adjacent sensors, the magnetic field excited by a coil could superimpose the magnetic field of another coil (crosstalk). On the other hand, coupling into another oscillatory circuitry tuned at a similar base frequency, *f*_0_, would mean a considerable power loss for the exciting coil. This is the basic principle of the so-called grid dip meter, a measuring instrument for obtaining the resonant frequency of radio frequency circuits [22].

**Figure 5.**
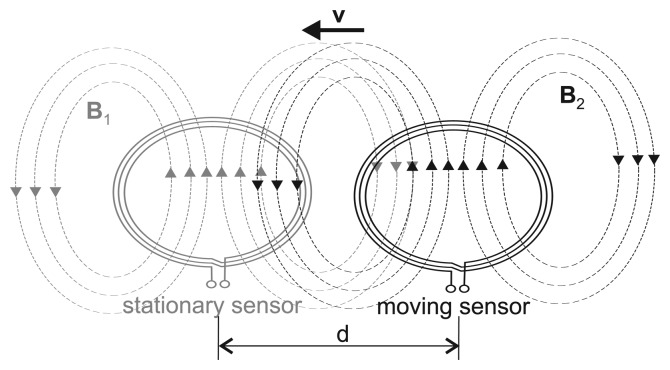
Measurement setup for the investigation of the mutual interaction between adjacent coils.

In rigid measurement systems, as presented in [15], the interaction between adjacent coils may only influence the offset of the signal. However, for the flexible sensor array presented here, mutual interaction plays an important role, since the distance and orientation between sensor coils may change during the measurement. To find the minimal (*i.e.*, non-interfering) distance between sensors for given coil parameters, the measurement setup shown in Figure 5 was used. Starting at a distance of 30 cm, a sensor coil (base frequency *f*_0,1_) was moved towards a second, stationary sensor coil (base frequency *f*_0,2_) while the sensor signal was measured. This measurement was conducted several times with varying base frequency difference Δ*f*_0_ = *f*_0,1_ − *f*_0,2_.

### 2.4. Monitoring of Respiration and Pulse

#### 2.4.1. Measurement Setup

The ability of the presented MAIN Shirt system to monitor cardiorespiratory activity was evaluated by measurements in volunteers. Eight healthy male volunteers were asked to wear the MAIN Shirt over one layer of cotton. They performed 150 s of normal breathing in a standing and in a sitting posture and a 20 s apnea phase in a standing posture. For pulse and respiratory references, the signals of a photoplethysmographic (PPG) finger clip sensor (ChipOx, Corscience GmbH & Co. KG) and a flow meter (Model 4040, TSI Incorporated, Shoreview, MN, USA) were recorded simultaneously.

Furthermore, to investigate the system's potential for pulse detection during normal breathing and motion, the volunteers performed additional 150-sec phases of normal breathing during slow walking. This measurement was done without the respiratory reference, since the flow meter has a high breathing resistance, which would disturb the subject and interfere with measurement, due to unnaturally augmented respiration. For pulse detection during breathing, the raw signal was processed by discrete wavelet transform (DWT) (described in Section 2.4.3.).

#### 2.4.2. Performance Metrics

To evaluate the signals derived by the MAIN Shirt at the different sensor positions, the following performance metrics were used:

- Signal-to-noise ratio (SNR): the ratio between the respiratory or cardiac signal and high frequency noise (≥ 20 Hz) in decibels.
- Signal-to-motion ratio (SMR): the ratio between the respiratory signal and signal content other than high frequency noise (presumably related to random body motion, manually extracted after visual examination).
- Coverage: the percentage of the measurement period during which the respiratory or cardiac cycles were detectable in the MAIN Shirt signal.
- Δ*f_cycle_*: the mean peak-to-peak frequency change in hertz of the sensor's oscillatory circuit (*i.e.*, signal output) during a respiratory or cardiac cycle.

Furthermore, after the calculation of the performance metrics listed above, the sensor with the highest coverage rate was chosen and compared with the corresponding reference device using Bland-Altman plots [23]; *i.e.*, breath-to-breath (*BrBr*) and heart beat-to-beat (*BB*) intervals were extracted from the MAIN Shirt, as well as from the reference signal, and the mean value of and the difference between each pair is shown in a scatter plot. Horizontal lines indicate mean difference and mean difference +/− 1.96 times the standard deviation (SD) (95 % confidence interval; CI).

#### 2.4.3. Signal Processing

For pulse detection during activity, data were processed by discrete wavelet transform (DWT). The use of DWT allows one to divide the sampled signal, *x*(*n*), into low frequency (*a_k_*(*n*)) and high frequency (*d_k_*(*n*)) components by bisection of its frequency range using low-, *g*(*n*), and high-, *h*(*n*), pass quadrature mirror filters [24]. This procedure is iterated using the low frequency component of the preceding iteration until a user-defined level, *k*, is reached. The form of the filter is called a wavelet. The original signal can be recomposed out of the *a_k_* and *d_k_* components without any loss of information by an inverse synthesis filter bank.

In the present study, we applied a DWT with the coiflet-5 wavelet until the sixth decomposition level. Afterwards, the signal was recomposed by setting all components (except *d*_5_ and *d*_6_) to zero. In this way, only the frequency range in which the pulse signal is expected (0.744–2.976Hz, *i.e.*, 44.6–178.6bpm) was considered, and all other frequency components were suppressed.

According to [25], the generation of pulse-related alarms within 10 s is sufficient for cardiac monitors and meters. Therefore, the signal was segmented into non-overlapping 10 s windows before the described DWT processing was applied. Afterwards, the beats (*i.e.*, peaks) were detected; the mean cardiac beat-to-beat length, *BB*_10_, for each time window was calculated, and the result with the lowest absolute value of the relative error was chosen.

## 3. Results

To investigate the sensors' characteristics (*i.e.*, the sensitivity to object distance and conductivity, measuring depth and mutual interaction), laboratory experiments were conducted (Section 2.3); the results are presented in Section 3.1.

Section 3.2 presents the results of the cardiorespiratory activity monitoring of the MAIN Shirt when worn by healthy volunteers (as described in Section 2.4).

### 3.1. Experimental Sensor Characterization

#### 3.1.1. Sensor Response and Measuring Depth

**Figure 6.**
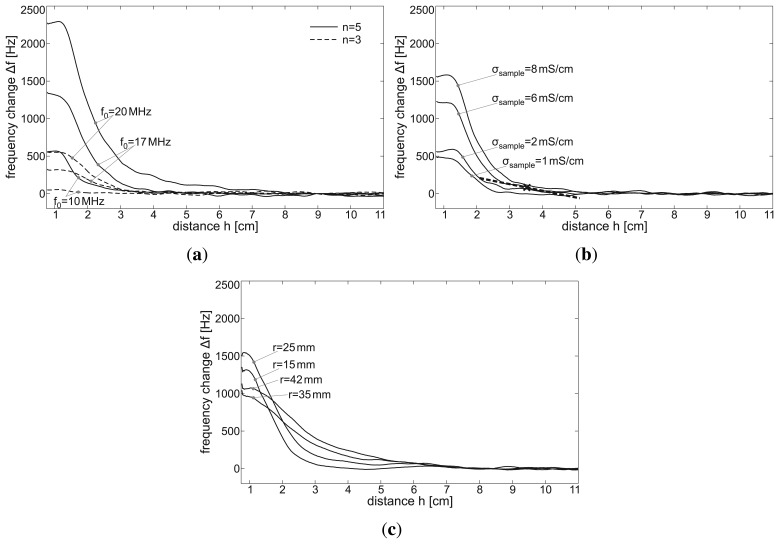
Results of the examination of sensor response and measuring depth. The signal output, Δ*f*, is presented in relation to the signal-to-object distance (height *h*). *f*_0_ is the tuned base frequency of the sensor, *σ_sample_* the conductivity of the sample object, *r* the radius of the sensor coil and *n* the number of windings. **(a)** *f*_0_ is varied, *σ_sample_* = 8 ms/cm, *r* = 30 mm; **(b)** *σ_sample_* is varied, *f*_0_ = 17 Mhz, *n* = 5, *r* = 30 mm; **(c)** *r* is varied, *σ_sample_* = 6 ms/cm, *f*_0_ = 20 Mhz, *n* = 5.

Based on the experiment described in Section 2.3.1., Figure 6a presents the signal output, Δ*f*, of the sensor caused by a conductive sample with conductivity *σ_sample_* = 8 ms/cm in relation to the sensor-to-object distance, *h*. This measurement was conducted with different tuned base frequencies, *f*_0_, and numbers of coil windings, *n*. Apparently, the higher the base frequency of the sensor, the higher the signal output. Furthermore, using coils with five windings yields higher frequency changes than using coils with three windings. The small oscillations in the decreasing tails are presumably caused by the swinging of the sample tank or by a non-smooth characteristic of the DC motor and its cable winch. To determine the maximum distance at which the detection of object motion is still possible, we define the measuring depth as the distance at which the signal-to-noise ratio becomes 3dB, *i.e.*, the signal magnitude (Δ*f*) caused by a conductive sample with *σ* = 8ms/cm at this distance is $\sqrt{2}$ times greater than the noise floor. Table 3 lists the measuring depth for the different *f*_0_ and *n*. Figure 6b presents the results of the same experiment conducted with a base frequency of 17 MHz, with five windings and different sample conductivities. As expected, the signal response increases with sample conductivity.

To evaluate the impact of organ displacement and organ conductivity on the sensor signal during a cardiac cycle, we examine the sensor's sensitivity at *h* = 3.5 cm for *σ_sample_* = 6 ms/cm and *f*_0_ = 17 MHz in more detail. This point is chosen based on magnetic resonance imaging (MRI) data from one of our monitored volunteers [26]. Using these MRI data, the distance between the heart wall and a sensor coil placed at position 1 (see Figure 2a) was determined to range approximately from three to 4 cm during a cardiac cycle. Furthermore, according to Table 2, the average conductivity of the heart during diastole and systole is assumed to change 6–8 ms/cm, respectively. Linearization at this point (as shown in Figure 6b, dashed line) yields a sensitivity on object motion of *S_motion_* = 86 Hz/cm, while the sensitivity on object conductivity at this distance is *S_cond_* = 28 Hz/(2ms/cm) = 14 Hz/(ms/cm). Given that these assumptions are valid, this would mean that during a heart beat, the impact of heart displacement on the sensor signal should be 3.1 times higher than that of heart conductivity changes.

Figure 6c shows that the measuring depth increases with the coil radius, which is in accordance with [27]. In contrast, when the sample comes into close vicinity of the coil, this relation changes, and coils with smaller radii can become more sensitive than coils with higher *r*.

#### 3.1.2. Mutual Interaction between Adjacent Coils

**Figure 7.**
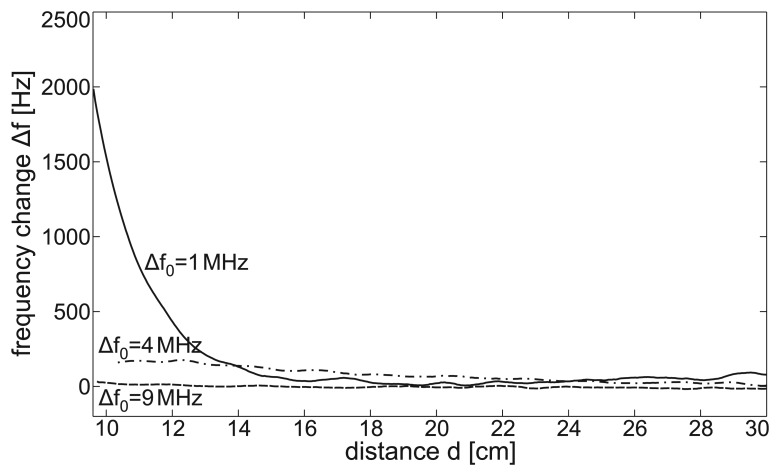
Results of the examination of the mutual interaction between adjacent sensor coils. The distance, *d*, between two coils is varied by moving one coil along a carriageway towards another one, whose signal output, Δ*f*, is plotted. Δ*f*_0_ denotes the difference between the coils' base frequencies.

Figure 7 shows the signal change of a sensor coil due to mutual interaction with another sensor coil moving towards it. The stationary sensor coil comprises one winding and is tuned at *f*_0, 1_ = 16 MHz. The moving coil comprises five windings and is tuned at *f*_0, 2_ = 17, 20 and 25 MHz, *i.e.*, Δ*f*_0_ = *f*_0, 2_ − *f*_0, 1_ = 1, 4 and 9 MHz. Both coils have a radius of *r* = 30mm. The distance, *d*, between the coils is measured between the coil centers.

Figure 7 shows that there is enormous mutual disturbance between the sensors with Δ*f*_0_ = 1 MHz, which reaches a signal magnitude of 2,000 Hz at a distance of 10 cm. This is similar to the magnitude caused by conductive samples directly above the sensor coil. By choosing higher differences in the tuned base frequencies of both coils (here, Δ*f*_0_ = 4 and 9 MHz), the mutual interaction can be considerably decreased.

### 3.2. Monitoring of Respiration and Pulse

#### 3.2.1. Respiration Monitoring

**Figure 8.**
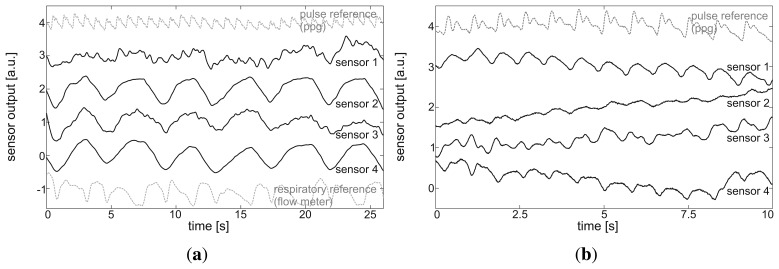
Representative excerpt of the MAIN Shirt's raw signal at each sensor position during **(a)** normal breathing and **(b)** apnea in a standing posture. For illustrative purposes, the signals are scaled to the same magnitude and shifted.

Normal breathing was performed by all subjects in both the standing and sitting posture for 120 s. Figure 8a shows a representative 25-second excerpt of the raw signal of the MAIN Shirt during normal breathing in a standing posture. In both postures, respiratory monitoring was easily achieved. Note that the respiratory reference signal presents lung flow (first derivative of lung volume) and, therefore, shows two maxima per respiratory cycle. To evaluate the signals derived by the MAIN Shirt at the different sensor positions, the performance metrics described in Section 2.4.2. were calculated.

**Figure 9.**
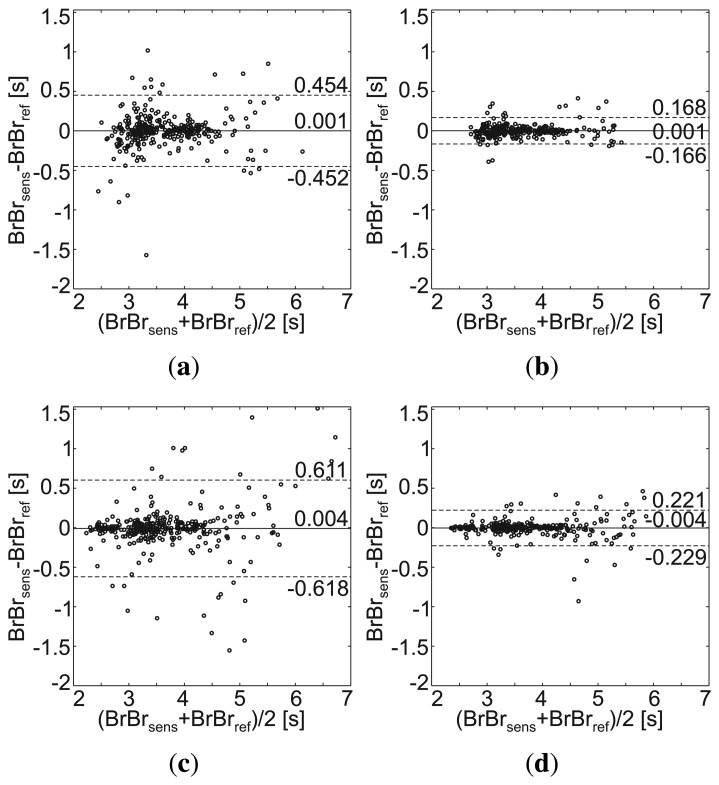
Bland–Altman plot of the breath-to-breath intervals, ***BrBr**_sens_* and ***BrBr**_ref_*, recorded by the MAIN Shirt and the reference sensor, respectively, during normal breathing in (**a**,**b**) standing and (**c**,**d**) sitting posture. (a,c) Non-smoothed values obtained from the raw signal; (b,d) values smoothed by a sliding average of three elements.

The results during normal breathing in a standing posture are presented in Table 4a. Sensors 3 and 4 (positioned at the abdomen and back, respectively) show better SNR and SMR than those positioned at the chest. Since sensor 4 performs with the highest coverage rate (99%), this sensor is chosen for the Bland–Altman plot presented in Figure 9a. Here, the lengths of the breath-to-breath period derived with sensor 4 (*BrBr_sens_*) are compared with those recorded with the reference sensor (*BrBr_ref_*). While the accuracy is sufficient (mean difference = 0.001 ms), the standard deviation (SD) of 0.231 s is relatively high. When the period lengths measured with the MAIN Shirt are smoothed by a three-element sliding average, the SD becomes smaller by a factor of 2.7 (SD = 0.085 s) (see Figure 9b).

In sitting posture, the signal quality of sensor 4 decreased significantly, while that of sensor 3 slightly increased (Table 4b). Therefore, sensor 3 was chosen for the Bland–Altman plots presented in Figure 9c and 9d. The results are similar to those acquired in the standing posture (Figure 9a and 9b).

#### 3.2.2. Pulse Monitoring

**Figure 10.**
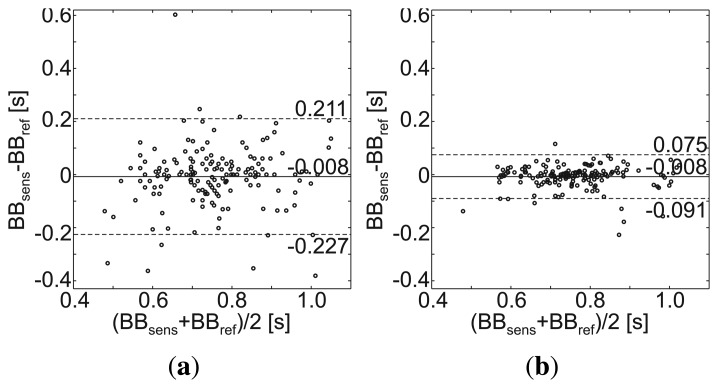
Bland–Altman plot of the beat-to-beat intervals, ***BrBr**_sens_* and ***BrBr**_ref_*, recorded by the MAIN Shirt and the reference sensor, respectively, during apnea. (**a**) Non-smoothed values obtained from the raw signal; (**b**) values smoothed by a sliding average of five elements.

During a 20-second apnea phase performed by each subject, the cardiac cycles were extracted and the performance metrics were calculated (described in Section 2.4.2.). Figure 8b shows a representative 10-second excerpt of the sensors' raw data, and Table 5 lists the performance metrics. However, sensor 3 (abdomen) showed a slightly higher SNR = 62.5 dB; sensor 1 (chest, left) was chosen for the Bland– Altman plot, due to its significantly higher coverage rate (85.1 %). The Bland–Altman plot in Figure 10a presents a slight inaccuracy (mean difference = −0.008 sec). Since the cardiac related signal content within the MAIN Shirt signal presented a variable phase shift, the standard deviation is relatively high (SD = 0.112 s). If the values are smoothed by a sliding average of five elements (as in Figure 10b), the SD can be decreased by a factor of 2.7 (SD = 0.042 s).

**Figure 11.**
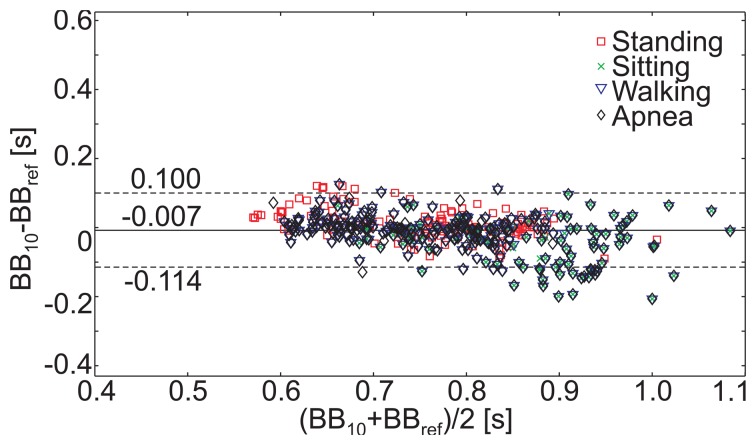
Bland–Altman plot of the beat-to-beat intervals, ***BrBr***_10_ and ***BrBr**_ref_*, recorded by the MAIN Shirt and the reference sensor, respectively, during different postures and movements. The MAIN Shirt data were processed as described in Section 2.4.3.

To investigate the system's potential for pulse detection during normal breathing and motion, the volunteers also performed 150 s phases of normal breathing during slow walking. The data were segmented into 10 sec windows, and the mean cardiac beat-to-beat lengths, *BB*_110_ were estimated, applying a discrete wavelet transform (as described in Section 2.4.3.). The absolute value of the relative error, *R_rel_*, is presented in Table 6 for each activity. The mean *R_rel_* is 5.1 %. The corresponding Bland–Altman plot can be seen in Figure 11, itemized for each activity. The mean difference is -0.007 s and the SD is 0.055 s.

## 4. Discussion

The results of the experimental measurements (described in Section 2.3.1.) show that the sensitivity of the magnetic induction sensors and, therefore, the measuring depth, depends on the tuned base frequency, *f*_0_, of the sensor and on the number of windings, *n*. The estimated ratio between the signal output due to heart motion and due to heart conductivity indicates a 3.1 times higher impact of heart motion compared with heart conductivity. This means that both effects have a range in the same order of magnitude and will measurably affect the signal. It has to be stated that the experimental results used for our estimation (Figure 6b) show some difference when compared with the results of another run with the same parameters (Figure 6a). Therefore, further investigations about the repeatability of the experiments would be helpful in future studies. Nevertheless, pulse measurements in healthy volunteers (Section 3.2.2.) show much higher signal outputs compared with data from our laboratory experiments. This could indicate that deformation or motion of the sensor coils due to the motion of the thoracic wall is the main influence.

As expected, the sensor sensitivity shown in Figure 6c increases with the radius of the coil; however, when the moving conductive sample comes in close vicinity of the coils (distance ≤ 2 cm), the smaller coils become more sensitive. This might be explained by the size of the sample tank (with a radius of 2.0 cm). For small coil radii and distances, the entire sensitive coil area is overlaid by the conductive sample tank, yielding higher frequency shifts than for larger coils, whose sensitive area is partly untouched by the sample in its front.

Monitoring the respiratory rate of all volunteers resulted in very high coverage rates and SNR without any signal processing (Table 4). Since sensors 3 and 4 are positioned at the abdomen and back, respectively, and are, therefore, more distant from the heart than sensors 1 and 2, they show a higher SMR. In a standing posture, the sensor at the back shows the best signal quality, whereas the sensor on the abdomen performed better in a sitting posture. This may be due to the usual variance in breath-related thoracic wall motion in the different postures [28]; however, the change in performance of the sensor on the subject's back could also be caused by the subject leaning against the chair's backrest. In contrast to its very high accuracy, the breath-to-breath lengths obtained by the MAIN Shirt showed a relatively high standard deviation from the true situation, which can be greatly reduced by smoothing over three samples. This deviation was probably due to the flat characteristic of the respiratory signal curve and a low sampling rate of 100 Hz impeding proper peak detection, as well as to the non-ideal reference (flow signal compared to volume signal).

Pulse monitoring during apnea is possible with the MAIN Shirt without further signal processing. The sensor beneath the left chest produced the best performance, due to its advantageous position. When applying the DWT-based algorithm described in Section 2.4.3., extraction of the beat-to-beat length in the 10-sec windows was possible, even during light activity, such as normal breathing and slow walking. However, the algorithm needs to be further enhanced by proper automatic selection of the sensor with the best signal quality.

Furthermore, the redesign of the MAIN Shirt based on the findings from the laboratory experiments seems advisable. To achieve higher measuring depths, higher coil radii, *r*, as well as higher base frequencies, *f*_0_, would be advantageous. Unfortunately, this design optimization is limited by the mutual interaction between coils (see Section 3.1.2.). Increasing the coil diameter will decrease the distance between the adjacent coils. In addition, it is not possible to tune all coils at the maximum base frequency, since there has to be a minimum difference in their base frequencies to suppress the “grid dip meter” effect. However, to enhance the sensitivity of sensor 1 around the heart wall distance, the coil parameters (*i.e.*, *r* and *f*_0_) of sensors 1 and 2 should be swapped. Moreover, each sensor slave could be supplied by a single battery and the communication wires between the slave and master unit could be replaced by wireless data transmission, extending the MAIN Shirt to a wireless body sensor network, as presented, for example, in [29]. However, this would have the adverse effect that each of the sensor slaves would require an individual battery power supply.

## 5. Conclusion

The system (MAIN Shirt) described here represents the successful implementation of a magnetic induction sensor array that can easily be integrated into textiles. The laboratory experiments confirmed the assumed physical principle. The sensors measure the conductivity distribution within an object and are sensitive to the displacement and the changes in the conductivity of organs. The impact of heart motion on the signal is estimated to be 3.1 times higher than the impact of heart conductivity changes during a cardiac cycle. Measurements with healthy volunteers have demonstrated the feasibility to reliably monitor cardiorespiratory activity. Respiratory monitoring is possible without further signal preprocessing, and the extraction of cardiac beat-to-beat lengths during light activity is feasible with simple signal processing based on discrete wavelet transform.

Due to its excellent characteristics of unobtrusive and easy integration into daily routines, the MAIN Shirt seems to be highly suitable for long-term home monitoring.

## Figures and Tables

**Table 1.** Specification of the sensor coils with radius *r*, number of windings *n*, base frequency *f*_0_, coil inductance *L_coil_* and oscillatory capacitance *C_osc_*.

**No.**	**Position**	***r*(mm)**	***n***	***f*_0_ (MHz)**	***L****_coil_***(*μ*H)**	***C****_osc_* **(pF)**
1	chest, left side, beneath	3.00	3	17	1.55	56
2	chest, right side, above	2.25	3	20	1.13	56
3	abdomen	2.25	3	20	1.13	56
4	back, right side	3.00	3	17	1.55	56

**Table 2.** Sample conductivity, σ*_sample_*, and the corresponding tissue types. All values apply for 10MHz [19–21].

*σ_sample_*	Tissue
1 ms/cm	bone, fat
2 ms/cm	lungs (inspiratory), skin (dry), spinal cord
4 ms/cm	lungs (expiratory), blood vessel, aorta, skin (damp)
6 ms/cm	muscle, heart
8 ms/cm	blood

**Table 3.** Measuring depth for different base frequencies, *f*_0_, and numbers of windings, *n*. (*r* = 30mm).

	**Measuring depth (cm)**	
*f*_0_	*n* = 3	*n* = 5
10 MHz	1.82	3.33
17 MHz	3.05	5.63
20 MHz	3.65	9.44

**Table 4.** Performance metrics of the different sensor positions during normal breathing in **(a)** a standing and **(b)** a sitting posture. Values are averaged for all subjects (the total signal length is 16 min). Δ*f_resp,cycle_* is the mean peak-to-peak frequency change of a respiratory cycle. SNR, signal-to-noise ratio; SMR, signal-to-motion ratio.

**Sensor No.**				
**Metric**	**1**	**2**	**3**	**4**
SNR (dB)	107.0	65.2	130.6	151.1
SMR (dB)	26.3	46.8	62.8	53.6
Coverage (%)	85.6	97.9	97.1	99.0
Δ*f_resp,cycle_* (Hz)	3,714.8	630.6	13,434.1	26,459.2

(a)					
**Sensor No.**				
**Metric**	**1**	**2**	**3**	**4**

SNR (dB)	98.6	61.4	138.1	137.9
SMR (dB)	36.0	44.0	54.3	28.2
Coverage (%)	94.1	94.8	100	88.6
Δ*f_resp,cycle_* (Hz)	8,086.3	4,470.6	66,088.8	16,737.2

(b)					

**Table 5.** Performance metrics of the different sensor positions during breath holding in a standing posture. Values are averaged for all subjects (the total signal length is 160 s). Δ*f_pulse,cycle_* is the average peak-to-peak frequency change of a cardiac cycle.

**Sensor No.**				
**Metric**	**1**	**2**	**3**	**4**
SNR (dB)	61.7	55.7	62.5	53.7
Coverage (%)	85.1	83.8	63.5	68.5
Δ*f_pulse,cycle_* (Hz)	602.5	725.6	974.9	779.0

**Table 6.** Absolute value of the relative error, *R_rel_*, of mean beat-to-beat lengths, *BB*_10_, after signal processing, as described in Section 2.4.3.

**Posture**	***R_rel_* (%)**
Standing	5.0
Sitting	5.8
Walking	4.2
Apnea	5.5
*Mean*	*5.1*
